# ctDNA and Adjuvant Therapy for Colorectal Cancer: Time to Re-Invent Our Treatment Paradigm

**DOI:** 10.3390/cancers13020346

**Published:** 2021-01-19

**Authors:** Mahendra Naidoo, Peter Gibbs, Jeanne Tie

**Affiliations:** 1 Peter MacCallum Cancer Centre, Department of Medical Oncology, Melbourne, VIC 3000, Australia; mahendra.naidoo@petermac.org; 2 Division of Personalised Oncology, The Walter and Eliza Hall Institute of Medical Research, Melbourne, VIC 3052, Australia; peter.gibbs@wehi.edu.au; 3 Western Health, Department of Medical Oncology, Melbourne, VIC 3021, Australia; 4 Faculty of Medicine, Dentistry and Health Sciences, University of Melbourne, Melbourne, VIC 3010, Australia

**Keywords:** ctDNA, adjuvant chemotherapy, colorectal cancer, biomarker, minimal residual disease

## Abstract

**Simple Summary:**

There is currently a lack of useful tests to detect microscopic residual disease in patients who have undergone surgery to remove their bowel cancer. This inability to identify patients with microscopic cancer could lead to over- and under-treatment with chemotherapy. Circulating tumour DNA (ctDNA) has shown significant promise to fill this gap to potentially personalize treatment after curative intent surgery allowing de-intensifying and intensifying of adjuvant therapies to reduce unnecessary toxicity of systemic therapy and also to hopefully cure more patients with ‘high risk of relapse’. This review article focuses on the current clinical use and future direction of ctDNA for early-stage bowel cancer.

**Abstract:**

Colorectal cancer (CRC) is one of the leading causes of cancer-related deaths worldwide. While there have been significant developments in the treatments for patients with metastatic CRC in recent years, improving outcomes in the adjuvant setting has been more challenging. Recent technological advances in circulating tumour DNA (ctDNA) assay with the ability to detect minimal residual disease (MRD) after curative intent surgery will fundamentally change how we assess recurrence risk and conduct adjuvant trials. Studies in non-metastatic CRC have now demonstrated the prognostic impact of ctDNA analysis after curative intent surgery over and above current standard of care clinicopathological criteria. This ability of ctDNA analysis to stratify patients into low- and very-high-risk groups provides a window of opportunity to personalise adjuvant treatment where escalation/de-escalation of adjuvant systemic therapy could potentially increase cure rates and also reduce treatment-related physical and financial toxicity. Emerging data suggest that conversion of ctDNA from detectable to undetectable after adjuvant chemotherapy may reflect treatment efficacy. This real-time assessment of treatment benefit could be used as a surrogate endpoint for adjuvant novel drug development. Several ctDNA-based randomized adjuvant trials are ongoing internationally to confirm the clinical utility of ctDNA in colorectal cancer.

## 1. Introduction

Colorectal cancer (CRC) is the third most common cancer worldwide and is the second leading cause of cancer-related deaths [1]. The total number of deaths is predicted to rise in rectal and colon cancer by 60% and 71.5% respectively by the year 2035 [2]. Although there have been significant advances in the treatment for patients with metastatic disease with median overall survival now exceeding 24 months [3,4,5], a cure remains elusive for the majority of patients. Early cancer detection and eradication of occult microscopic disease with adjuvant treatment in non-metastatic or early-stage cancer therefore represent the two most substantial opportunities to achieve a cure and improve survival [6,7,8].

The current standard of care for early-stage CRC is surgery, and if indicated, followed by up to six months of adjuvant chemotherapy. While the benefit of adjuvant chemotherapy has been unequivocally established in stage III colon cancer [9,10], the role of adjuvant chemotherapy in stage II colon cancer remains the subject of much debate and is not recommended for all patients [11]. Clinical guidelines currently recommend that adjuvant chemotherapy should be considered in stage II CRC with high-risk clinicopathological features (e.g., T4 extension, lymph node sampling < 12, lymphovascular invasion), following the rationale that patients with a higher risk of recurrence may benefit from adjuvant chemotherapy [12,13]. However, this approach of selectively treating stage II patients with poor prognostic features has not conclusively been shown to improve overall survival [14,15]. To this end, better prognostic and/or predictive biomarkers are needed clinically to help identify the patients who will benefit most from adjuvant therapy.

In stage III CRC, the added absolute benefit of adjuvant chemotherapy is typically quoted to be around 12% with single agent fluoropyrimidine with an additional 6% benefit in combination with oxaliplatin [10,16,17,18]. We have to be mindful that these established benefits were based on historical clinical trial data predating modern-day surgical techniques and pre-operative staging with contrast-enhanced CT scan or positron emission tomography. If the absolute risk of recurrence is lower today due to better surgery and stage migration with better imaging, it is likely that the absolute gain from adjuvant chemotherapy especially oxaliplatin, may be less than they once were. This, along with the risk of long-term peripheral neuropathy associated with oxaliplatin [19,20,21], have motivated an unprecedented international effort to examine a de-escalated treatment approach with a shorter duration (three months) of adjuvant chemotherapy compared to the standard six months of treatment in stage III CRC [22]. The latest overall survival update presented at the 2020 ASCO (American Society of Clinical Oncology) Annual Meeting [23] observed minimal difference (0.4%) between the two groups, setting three months of adjuvant oxaliplatin-based combination treatment as the new standard for stage III CRC, especially those with clinically low-risk disease. Importantly, results from the IDEA (International Duration Evaluation of Adjuvant Therapy) meta-analysis reminds us of the need to continually re-evaluate the risk–benefit ratio of our current treatment recommendation and provides reassurance that less treatment is not necessarily detrimental to patient outcome.

Another challenge beyond the imprecision of patient selection for adjuvant therapy, is the lack of progress with better treatment beyond oxaliplatin and fluoropyrimidine in the past 16 years. Agents that have shown efficacy in the metastatic setting (irinotecan, bevacizumab, and cetuximab) have thus far failed to demonstrate significant survival benefit compared to fluoropyrimidine or oxaliplatin-based combination treatment in eight randomised trials [24,25,26,27,28,29,30]. This challenge to detect a benefit with new adjuvant therapy may in part be due to the improvement in recurrence risk, hence low event rate, over time with better multidisciplinary medical care as discussed previously. Additionally, the current model of adjuvant clinical trial based on an undifferentiated pathological staging alone (e.g., stage II and III) and an infrastructure which was developed over 50 years ago is highly inefficient, requiring many thousands of patients over a long period of time to capture recurrence and overall survival events. In this regard, biomarkers that could allow prognostic enrichment for high-risk patients and provide early read-out of adjuvant treatment efficacy could expedite novel drug development in the adjuvant setting.

One promising biomarker that has received significant attention in recent years is circulating tumour DNA (ctDNA). ctDNA are DNA fragments that are released by dying cancer cells into the bloodstream and in theory should contain genetic and epigenetic changes identical to the cancer cells they originated from. There are accumulating evidence that ctDNA analysis can be used to evaluate the presence of minimal residual disease (MRD) and predict recurrence in the post-operative setting. For those receiving adjuvant treatment, the non-invasive and dynamic nature of this marker may also reflect adjuvant chemotherapy efficacy in real-time. This review will summarize the current evidence on ctDNA as a MRD marker in colorectal cancer and how this could be used to fill the clinical gaps discussed above, including several ongoing ctDNA-based randomized adjuvant trials aiming to demonstrate its clinical utility.

## 2. Circulating Tumour DNA and Minimal Residual Disease

ctDNA is derived from cancer cells and released into the blood stream as a result of tumour cell necrosis [31]. This is distinguished separately from circulating free DNA (cfDNA) which is derived from non-cancer cells, free of molecular pathological alterations (e.g., somatic re-arrangements) and consists of longer base pair lengths compared to ctDNA [32]. ctDNA was first described in 1948 by Mendal and Metais [33] but the relevance to clinical application only became apparent in 1994 when RAS mutations were identified in ctDNA [34]. ctDNA represents only a small fraction of the total cfDNA, but this fraction is highly variable, ranging from less than 0.1% to greater than 10% depending on tumor stage, disease burden, biologic shedding or proliferation, and anatomic factors such as disease site [35,36]. Once in the circulation, ctDNA is cleared rapidly from the bloodstream, with a half-life of approximately 2 h [37], offering a real-time dynamic measure of tumor burden.

ctDNA is now found in both early-stage and metastatic disease across different solid tumour types, but the detection rate varies between tumour types and different stages of the same tumour type [35]. ctDNA has been shown to correlate with disease burden and treatment response in metastatic CRC [38,39]. Clinical use of ctDNA in the metastatic CRC setting includes genomic profiling to guide targeted therapy (e.g., identifying RAS mutations in guiding decision-making for anti-EGFR therapy), tracking resistance mechanisms, and timing of anti-EGFR rechallenge [6,7,8].

Minimal residual disease (MRD) is a term used to describe persistent micro-metastatic disease after definitive treatment (e.g., resection) of primary malignancy and/or completion of adjuvant systemic therapy thereafter. Importantly, this represents an occult state of disease that is not detectable by conventional imaging modalities or blood tests. The prognostic role of ctDNA-based MRD detection is now established in various haematological diseases [40,41,42,43,44,45] where MRD has now been incorporated into standard clinical guidelines [46]. In fact, the expanded U.S. Food and Drug Administration (FDA) approval of blinatumomab in March 2018 to treat adults and children with B-cell precursor acute lymphoblastic leukemia who are in remission, but still have MRD, was the first time the FDA used MRD as a biomarker for a regulatory decision. Despite this success in haematological malignancies, until recently, the clinical validity of ctDNA-based MRD detection in solid tumours was limited by the technical challenge of reliably detecting and quantifying these rare tumour DNA amongst the several thousand genome equivalents of DNA that are present in 1 mL of circulating plasma (typically <0.01% of total cfDNA).

Advances in several molecular techniques allowing high-sensitivity ctDNA analysis has sparked recent interest in pursuing the clinical role of ctDNA for MRD detection across various tumour types [47]. ctDNA detection methodologies will not be the focus of this review but we would like to refer the readers to several excellent papers which have reviewed this topic in detail [48,49,50,51]. Broadly speaking, there are two approaches to ctDNA analysis for MRD detection following curative intent treatment in early-stage cancer: tumor-informed vs. tumor-agnostic approaches. In the tumor-informed approach, somatic mutations are first identified in an individual patient’s tumor tissue via targeted sequencing or whole exome sequencing, followed by targeted sequencing of plasma DNA using a personalized assay. Several tumor-informed personalized ctDNA assays have been developed (e.g., SafeSeqS, CAPP-Seq, Tam-Seq, TARDIS, Signatera, ArcherDX PCM, Radar) with limits of detection as low as 0.01% variant allele frequency (VAF) [52,53,54,55,56,57,58]. For the tumor-agnostic approach, ctDNA analysis is performed without prior knowledge of a patient’s tumor mutation profile and often includes broad panel-based sequencing or methylation assay (e.g., Guardant Health’s ‘LUNAR’ assay). Beyond NGS-based technique, another sensitive mutation-based ctDNA analysis method includes droplet digital PCR (ddPCR) [59]. However ddPCR assays are limited to specific single mutations or sets of highly related mutations at the same locus [60]. The advantages of the tumor-agnostic approach include its faster turn-around time, lower cost, and ability to detect emerging resistant mutations. However, the trade-off for not requiring tumor tissue is potentially a lower sensitivity for detecting the low level of ctDNA in the MRD setting. Though more resource-intense, the tumor-informed approach, especially where multiple personalized variants are tracked simultaneously in the plasma, offers the highest analytical sensitivity and is particularly well-suited for MRD detection and recurrence monitoring.

The currently approved FDA ctDNA clinical tests for metastatic disease are: ‘FoundationOne CDx’, ‘Praxis Extended RAS panel’, ‘Cobas KRAS Mutation Test’, ‘therascreen KRAS RGQ PCR Kit’, ‘Dako EGR pharmDx Kit’, and ‘therascreen BRAF V600E RGQ PCR Kit’. For MRD detection, the Signatera assay is approved for colorectal cancer, and the ClonoSeq assay for multiple myeloma, acute lymphoblastic leukemia, and chronic lymphocytic leukemia [61].

## 3. ctDNA and MRD Detection in Colorectal Cancer

The first evidence of ctDNA’s potential as a marker of MRD came from a study conducted at Johns Hopkins in 2008 involving 18 patients with resected colorectal liver metastases [37]. Using the BEAMing (beads, emulsion, amplification and magnetics) assay, the study demonstrated that ctDNA levels declined precipitously after resection of all visible tumours but remained detectable at first follow-up visit in 12 patients; all but one had experienced recurrence. In contrast, none of the four patients with undetectable ctDNA at first follow-up visit experienced recurrence. This result inspired subsequent clinical validation of ctDNA as a MRD marker in non-metastatic CRC.

Completed ctDNA studies in the non-metastatic setting have thus far been restricted to non-interventional studies (i.e., observation of ctDNA results without active escalation/de-escalation of treatment depending) of which the key studies are summarized in Table 1. 

A seminal study including 230 patients with stage II colon cancer [62] was among one of the largest and earlier studies which demonstrated the clinical validity of ctDNA (using the tumour-informed Safe-SeqS assay) in the adjuvant setting. In the 178 patients not treated with adjuvant chemotherapy, the study demonstrated that the presence of ctDNA 4 to 10 weeks after surgery predicted a very high risk of recurrence with an estimated 3-year recurrence-free survival (RFS) of 0%, whilst those with undetectable ctDNA after surgery has a 3-year RFS of 90% (hazard ration (HR), 18; *p* < 0.001). In patients treated with chemotherapy, the presence of ctDNA following completion of chemotherapy was also associated with an inferior recurrence-free survival (HR, 11; *p* = 0.001). The study also showed superiority of ctDNA over CEA (carcinoembryonic antigen) as a biomarker for detecting radiological recurrence; ctDNA was positive in 85% vs. 41% CEA elevation (*p* = 0.003) at time of radiological recurrence. A further study using the Signatera assay [56] in 130 patients across stages I–III similarly showed that post-operative ctDNA-positive patients were seven times more likely to relapse than ctDNA-negative patients (HR, 7.2; *p* < 0.001).

There are a couple of studies which included stage III patients exclusively [64,66]. The first of these is an observation cohort involving 96 patients treated with adjuvant chemotherapy. The post-operative ctDNA detection rate was 21% and ctDNA detection was associated with a significant inferior recurrence-free survival (HR, 3.8; 95% confidence interval (CI), 2.4–21.0; *p* < 0.001). This prognostic impact was independent of standard clinicopathological criteria. Importantly, ctDNA remains detectable at the end of chemotherapy in 17% of cases with an estimated 3-year RFS of 30% compared with 77% in those whose ctDNA were negative after treatment (HR, 6.8; 95% CI, 11.0–157.0; *p* < 0.001). To date, the largest reported ctDNA series is a retrospective analysis of 805 patients with stage III colon cancer enrolled in the IDEA-France phase III randomized trial [64] which investigated the outcome of 3 vs. 6 months of adjuvant oxaliplatin-based chemotherapy. Using a tumour-agnostic plasma only methylation assay, post-operative (post-op) ctDNA detection rate was 13.5%. The study has similarly demonstrated that positive post-op ctDNA was an independently prognostic biomarker and perhaps more importantly, also showed that patients with ctDNA-positive disease benefited more from 6 months of adjuvant treatment than those with ctDNA-negative disease.

The ultimate utility of ctDNA is to assess adjuvant treatment efficacy. If clinicians are able to identify which adjuvant therapy is effective during such treatment, as indicated by reduction and subsequent negative ctDNA status, there is a potential to de-escalate toxic therapy. Conversely if adjuvant therapy is not effective at eliminating ctDNA, then switching to alternative therapy including novel drugs may be warranted. Table 1 identifies prospective trials that monitored ctDNA after therapeutic interventions including primary resection (surgery) and during/after adjuvant chemotherapy. It has been shown across both stage II and III colon cancer that ctDNA positivity after adjuvant treatment completion was associated with poorer RFS [56,62,66]. These data suggest that persistent detection of ctDNA post-treatment reflects presence of micrometastatic disease, which ultimately is the source of clinical recurrence.

Collectively, several trials have now consistently demonstrated the prognostic value of post-op and post-treatment ctDNA assessment in various stages of non-metastatic CRC. Of note, the ctDNA detection rates and prognostic impact vary across studies due to variations in the disease stages included in the studies, ctDNA assays, and pre-analytic variables, such as plasma volume assessed and timing of blood collections.

## 4. ctDNA and Surveillance in CRC

The goal of surveillance is to detect early disease relapse and intervene with early local therapy or systemic treatment as clinically indicated to improve survival. The current ASCO CRC surveillance guidelines recommend a combination of serial CEA, clinical examination, CT imaging, and endoscopic examination [72] over a 5-year period to detect early relapse. However, there is no convincing evidence that these or more intensive surveillance result in improved overall survival [73,74,75].

The current standard of care surveillance biomarker used is CEA is limited in both sensitivity and specificity [76,77,78]. In an aforementioned ctDNA trial in Table 1 [62], 85% of patients were ctDNA-positive at the approximate point of confirmed radiologic recurrence, however CEA was only elevated in 41% of patients. Therefore, ctDNA appears to be a more sensitive measure of radiological recurrence. Table 1 also summarises significant lead times where reported from ctDNA positivity to radiological recurrence. Serial monitoring during post-treatment surveillance also appears to predict relapse with significant lead-time over serological CEA monitoring, ranging from 100 days to 8.7 months across different studies. These studies tested ctDNA at different time points and did not perform imaging with each ctDNA analysis, likely accounting for the variation in lead-time. Despite this, these studies illustrate that earlier detection of recurrence may be possible with incorporation of longitudinal ctDNA testing into surveillance programs. However, whether bringing forward the diagnosis of recurrence impacts survival outcomes is a key question to be answered by future studies.

## 5. ctDNA-Based Randomised Interventional Adjuvant Trials

Despite the undeniable prognostic implication of ctDNA detection immediately after curative intent surgery or after completion of standard adjuvant therapy, the clinical utilities of ctDNA analysis along the adjuvant treatment paradigm remains to be proven. How ctDNA can be used to guide adjuvant therapy and improve patient outcomes, both in terms of improving recurrence-free survival and reducing physical and financial toxicities, are the subjects of investigation in multiple ongoing ctDNA-based randomized clinical trials listed in Table 2. As such, we caution the premature adoption of ctDNA analysis into routine clinical practice until read-outs from these randomized trials have confirmed its utility.

**Figure 1:**
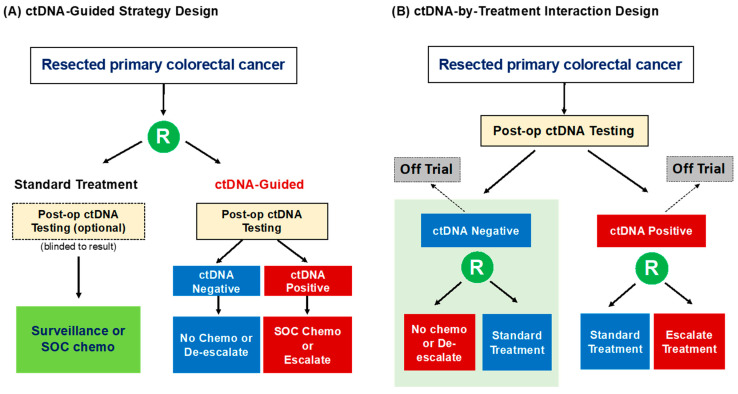
ctDNA-based randomised trial designs to demonstrate the clinical utility of ctDNA testing in the adjuvant setting.

Broadly speaking the clinical benefit of ctDNA analysis in the adjuvant setting can be assessed with two types of biomarker-based randomized trial designs (Figure 1) as previously described by Sargent et al. [87]. The “ctDNA-Based Strategy” design aims to compare the outcomes of a ctDNA-guided approach (experimental) with our standard non-ctDNA-guided approach (control). With this design, ctDNA testing can be limited to the ctDNA-guided cohort only or can be performed on all enrolled patients with the control group being blinded to the result. The treatment options for the ctDNA-positive and -negative patients in the ctDNA-guided arm will depend on the disease stage being studied and the current standard-of-care treatment, but would usually include an escalated treatment for the ctDNA-positive group and a de-escalated regimen for the ctDNA-negative group. The clinical value of ctDNA is assessed by comparing the outcome of all of the patients in the ctDNA-guided arm to that of all of the patients in the non-ctDNA-guided arm. For example, the Australian DYNAMIC trial randomises patients with resected stage II colon cancer to either a “standard-of-care” approach where the use of adjuvant chemotherapy or not is decided by the treating clinician based on conventional clinicopathological features; or a “ctDNA-guided” strategy where patients with a positive post-op ctDNA result will be treated with adjuvant chemotherapy (FOLFOX/CAPOX or single agent fluoropyrimidine) while those with a negative post-op ctDNA result will be managed by observation only. The DYNAMIC trial aims to demonstrate that a “ctDNA-guided” strategy will reduce the proportion of patients with stage II colon cancer requiring adjuvant chemotherapy without compromising recurrence-free survival when compared to our current non-ctDNA-guided approach. The DYNAMIC-III study is a similar strategy trial examining the value of ctDNA-guided de-escalation or escalation adjuvant chemotherapy (including FOLFOXIRI) approach in stage III colon cancer. The UK-based TRACC study is a randomized ctDNA-based strategy trial embedded within an observational study enrolling patients with high-risk stage II or stage III colon cancer. This study will test a “ctDNA-guided” de-escalation and subsequent escalation approach in patients with an initial negative post-op ctDNA, whereby a repeat ctDNA analysis performed at 3 months will determine if the initial de-escalated treatment (no chemotherapy or 3 months of capecitabine) should be escalated to 3 months of CAPOX.

In contrast, in the “ctDNA-by-Treatment Interaction” design, all patients undergo ctDNA testing and patients in one or both marker groups (ctDNA-positive or ctDNA-negative) are randomly assigned to two different interventions. The study could include both ctDNA-positive and -negative patients which will likely make it an impracticably large and costly study to conduct, or simply focus on the biomarker group of interest while the other group is treated off trial. Examples of “ctDNA-by-Treatment Interaction” design where the primary interest is in stage II colon cancer patients with post-op ctDNA-positive result include the NRG GI-005 COBRA, CIRCULATE-AIO, and CIRCULATE-PRODIGE 70 studies. The primary aim of all three trials is to demonstrate a recurrence-free survival benefit in patients with positive post-op ctDNA treated with adjuvant chemotherapy (FOLFOX/CAPOX or capecitabine) compared to observation alone. On the other hand, the Japanese VEGA study will explore the non-inferiority of a de-escalation approach in post-op ctDNA-negative patients by randomizing high-risk stage II and low-risk stage III patients with a post-op ctDNA-negative result to either 3 months of CAPOX (control) or surveillance (experimental).

Arguably, one of the most exciting clinical utilities for ctDNA is the potential to expedite novel drug development in the adjuvant setting, not only by enriching for high-risk patients, but also to provide an early read-out of treatment efficacy with serial ctDNA measurements. For patients with post-op ctDNA-positive disease, the magnitude of benefit from standard adjuvant chemotherapy remains to be confirmed in several of the ongoing randomized trials mentioned above. Until then, clinicians would logically be more comfortable exploring novel therapy in patients who remain at high risk for recurrence after completion of standard-of-care adjuvant chemotherapy; that is, in patients whose ctDNA remain positive or detectable at the end of treatment. Examples of this so-called “ctDNA-Enriched Second-Line Adjuvant Therapy” trial design include the US-based ACT-3 study and the Japanese ALTAIR trial. In the ACT-3 trial, patients who are ctDNA-positive after completion of 3–6 months of adjuvant FOLFOX or CAPOX are randomised to surveillance (control) or several molecularly-stratified “second-line” adjuvant therapies (experimental) including FOLFIRI (for microsatellite stable and BRAF wild-type tumours), encorafenib/binimetinib/cetuximab (for BRAFV600E mutant tumours), and Nivolumab (for microsatellite unstable tumours). The ALTAIR study will assess the efficacy of second-line adjuvant trifluridine/tipiracil in patients with ctDNA that remain positive after 3 months of adjuvant CAPOX. The PEGASUS study (NCT04259944) is a Italian/Spanish non-randomised ctDNA-guided interventional trial to explore the feasibility of a ctDNA-guided treatment strategy which also includes the use of second-line adjuvant FOLFIRI in patients with persistently positive ctDNA after standard CAPOX treatment [88]. One potential challenge with the second-line adjuvant therapy approach may be the relatively short time interval between ctDNA detection at the end of treatment and clinical recurrence, limiting the opportunity for therapeutic intervention. For studies exploring second-line novel adjuvant therapy, it is therefore imperative that a baseline re-staging imaging (e.g., PET/CT) is performed at enrollment to exclude clinically detectable metastatic disease in patients with persistently positive ctDNA after standard adjuvant chemotherapy.

Finally, the added value of ctDNA-guided surveillance strategy compared to standard CT imaging-based surveillance in high-risk stage II or stage III colorectal cancer will be addressed by the Danish IMPROVE-IT2 trial [86]. In the experimental arm, ctDNA analysis will be performed every 4 months for 24 months and a PET/CT will be performed if ctDNA becomes positive during surveillance. Patients in the control arm will receive the current Danish surveillance strategy which includes 12-month and 36-month CT scans. The primary endpoint of this study is the fraction of patients with recurrence receiving intended curative or local metastasis-directed treatment.

## 6. Challenges and Future Directions

While significant progress has been made towards the clinical translation of ctDNA, with results from ctDNA-based randomized trials maturing in the next few years, there are several challenges and questions that should be addressed in ongoing and future observation and interventional studies.

A major concern with MRD detection for solid tumours is the analytical sensitivity of the ctDNA assays in the post-op setting, in other words, the false negative rate. Even with advances in ctDNA assay where a limit of detection as low as 0.01% can now be achieved with tumour-informed ctDNA approaches, false negative results can still occur due to biological factors such as low DNA shedding tumours, mucinous histology, and anatomical location of the occult micrometastatic disease. Several studies in metastatic CRC have shown that patients with peritoneal, nodal, and lung only metastases are more likely to have undetectable ctDNA in plasma compared to patients with liver metastases [6,7,8,36]. Similarly, in a recent pooled analysis of 485 non-metastatic CRC cases, a false-negative ctDNA test at the immediate post-op timepoint is more commonly observed in patients who subsequently experienced loco-regional relapse alone (such as peritoneal or omental relapse) compared to those with distant relapse [89]. Studies in non-metastatic CRC that have collected pre-op plasma samples while the primary tumour is still in situ have reported ctDNA detection rates of up to 89% [56,65,68]. Thus, one potential strategy to reduce false negative post-op ctDNA results secondary to low-shedding tumours is to exclude the small proportion (~10%) of patients with a negative or undetectable pre-operative ctDNA from MRD assessment. However, we also need to be mindful of the additional resource required and practicality of collecting pre-op blood samples on all non-metastatic CRC patients given that pre-op imaging-based tumour staging may not reliably reflect pathological staging. For example, if pre-op blood samples are to be collected on all patients with non-metastatic CRC for a clinical trial that will only be enrolling pathologic stage III or high-risk stage II colon cancer, potentially up to half of the pre-op blood samples will not be used for study purpose. Additionally, the biology of shedding vs. low-shedding tumours, both in terms of prognosis and chemosensitivity, requires further elucidation and may impact the utility of pre-op ctDNA testing. If low-shedding tumours are associated with a good prognosis and contribute minimally to the post-op false negative cases, then the added value of pre-op ctDNA assessment will likely be trivial. It is noteworthy that other emerging circulating analytes such as DNA methylation and microRNA are currently in development and may be able to overcome or complement the analytical sensitivity limitation of mutation-based ctDNA testing [90,91].

Beyond assay sensitivity, the next relevant question one should ask is the optimal timing of blood collection after surgery to detect MRD and inform adjuvant therapy decision. It is well known that trauma such as that induced by surgery can increase the release of total cell-free DNA or wild-type DNA into the plasma, masking the detection of tumour DNA. It has been shown that total cell-free DNA levels remain elevated for up to 4 weeks after colorectal cancer surgery with consequential higher ctDNA detection rate for blood samples collected after 4 weeks of surgery compared to that from an earlier timepoint [92,93]. Another variable to consider is the progression of MRD over time, where one would reasonably expect to have a greater proportion of patients having detectable ctDNA with increasing time from surgery, meaning patients with an initial very low “tumor burden” and minimal release of ctDNA would eventually have a sufficient amount of ctDNA above the detection threshold with serial sampling. Consistent with this, it has been shown that patients with an initially negative post-op ctDNA, who later develop distant recurrence, will typically have ctDNA detectable on serial sampling several months before clinically detected disease [56,62,94]. One approach to sampling for MRD assessment would be to perform an initial blood draw at 4 weeks after surgery as a compromise between avoiding the immediate post-operative period and maximizing the time for ctDNA analysis (anticipating a turn-around time of 2 weeks for plasma analysis) to allow for adjuvant chemotherapy to be commenced within the standard 6- to 8-week post-op window. Those with an initial negative ctDNA could undergo further serial sampling at regular intervals (e.g., 3-monthly) and adjuvant treatment initiated or escalated if and when ctDNA becomes detectable. This strategy is currently being investigated by the UK TRACC study.

Studies to date of non-metastatic CRC have typically dichotomized ctDNA status into detectable (positive) vs. undetectable (negative). Today, ctDNA assays can reliably quantify ctDNA level and this assessment of micro-metastatic disease burden may further stratify prognosis or even predict the likelihood of adjuvant chemotherapy eradicating MRD and preventing cancer recurrence. Our recent pooled analysis observed an exponential increase in recurrence risk with rising ctDNA mutant allele fraction (MAF) and interestingly also found a smaller magnitude of benefit from adjuvant chemotherapy for patients with ctDNA MAF level higher than 0.046% [89]. Those with MAF higher than 0.046% remain at a very high risk of recurrence, despite adjuvant therapy, and represent a patient cohort where the most aggressive available treatment strategy including enrollment in clinical trials to explore novel therapy should be pursued. Given the retrospective nature of the analysis and the heterogeneity in disease stage and treatment included in the analysis, these observations can only be considered hypothesis generating at this stage and will require further validation in larger, more well-defined, patient population.

Over the years, several tumour tissue-based prognostic biomarkers, such as gene signatures and Immunoscore, have also been developed for early stage CRC [95,96,97]. The consensus Immunoscore assay is a well-validated complex scoring system summarising the density of CD3+ and CD8+ T-cell effectors within the tumour and its invasive margin using image-analysis software. Stage I to III CRC with high Immunoscore has been shown to be associated with lower recurrence risk than tumours with low Immunoscore (HR, 0.20; *p* < 0.0001) [97]. More specifically for patients with stage III disease, a recent retrospective series including patients untreated and those treated with a variety of adjuvant chemotherapy regimens reported 3-year recurrence-free rates of 56.9% and 76.4% in patients with low and high Immunoscores, respectively (HR, 0.48; *p* < 0.0003) [98]. A separate analysis in the high-risk stage II (T4N0) subset demonstrated a 5-year recurrence rate of 84.6% in patients with intermediate/high Immunoscore and 46.3% in patients with low Immunoscore [99]. While Immunoscore could help refine the prognosis of non-metastatic colon cancer patients in conjunction with the TNM staging and help guide adjuvant chemotherapy decision in stage II disease, its role in predicting chemotherapy benefit is currently uncertain and its utility should be investigated further in the context of a randomized trial [12]. In this regard, future research should explore opportunities to further refine recurrence risk assessment for stage II and III colon cancer by combining ctDNA analysis and Immunoscore or other methods of assessing tumour infiltrating lymphocytes (TILs).

## 7. Conclusions

Without question, the ability of current state-of-the-art circulating tumour DNA assays to directly assess MRD after curatively intent surgery has the potential to fundamentally change the adjuvant treatment paradigm for non-metastatic CRC. Not only will post-op ctDNA analysis offer more precise recurrence risk stratification in addition to pathological staging, potentially allowing intensity and duration of adjuvant therapy to be tailored based on ctDNA result, but serial ctDNA analysis may also provide an early real-time read-out of adjuvant treatment efficacy, improving the efficiency of adjuvant trial and novel drug development. While there are ongoing parallel efforts by clinicians and scientists to demonstrate the clinical utilities of ctDNA and to improve the sensitivity of ctDNA assay, researchers should also seek to standardize ctDNA assay characteristics and pre-analytical variables to ensure the reproducibility of testing results. Conducting health economic analysis of ctDNA-guided adjuvant strategy, involvement of other key stakeholders such as consumers with patient preference studies, and early engagement with regulatory agencies, are all critical steps towards clinical implementation of ctDNA.

## Figures and Tables

**Table 1:** Completed observational ctDNA studies in non-metastatic/oligometastatic CRC.

Reference	No. of Patients	Stages Evaluated	Method for ctDNA Analysis	Adjuvant Chemo Given	Key Results	% of Patients ctDNA Positive
Tie et al., 2016 [62]	230	II	Safe-SeqS	23%	In patients not treated with adjuvant treatment, presence of ctDNA after surgery was associated with an inferior recurrence-free survival (HR, 18; *p* = 0.001) 85% of patients were ctDNA-positive up to or at the time of radiologic recurrence, CEA was only elevated in 41% of patients. The median lead time from ctDNA detection to radiological recurrence was 167 days; range 81–279 days	Post-op: 7.9% Post-Treatment: 11% Surveillance: 11.7%
Reinert et al., 2019 [56]	130	I–III	Signatera	62%	Post-op ctDNA-positive patients were more than 7 times more likely to experience disease recurrence than ctDNA-negative patients (HR, 7.2; *p* < 0.001) Lead time to detect disease recurrence compared with standard surveillance: Mean 8.7 months; range 0.8–16.5 months	Post-op: 10.6% Post-Treatment: 12% Surveillance: 20%
Schøler et al., 2017 [63]	45	I–IV	ddPCR	36.8%	Longitudinal samples from 27 patients revealed ctDNA detection postoperatively in all relapsing patients (n = 14), but not in any of the non-relapsing patients. Of 21 patients treated for localised disease, all 6 ctDNA-positive patients (within 3 months of surgery) relapsed compared with 4 of the remaining patients (HR, 37.7; 95% CI; 4.2–335.5; *p* < 0.001). Time to detect disease recurrence of standard surveillance: Median lead time of 9.4 months, ranging from 0.4 to 14.9 months	Post-op: 28.6% (stages I–III) Post-Treatment: not reported Surveillance: not reported
Taieb et al., 2019 [64]	805	II–III	ddPCR (2 methylation markers)	All patients	2-year DFS was 64% vs. 82% in ctDNA-positive and -negative patients, respectively (HR, 1.75; 95% CI, 1.25–2.45; *p* < 0.001). Post-surgical plasma ctDNA predicted metastatic relapse a median of 10 months before recurrence was visible on radiological scans (HR, 11.33; *p* = 0.0001	Post-op: 13.5% Post-Treatment: not reported Surveillance: not reported
Tie et al., 2019 [65]	159	Locally advanced	Safe-SeqS	35.8% patients	Significantly worse recurrence-free survival was seen if ctDNA was detectable after chemoradiotherapy (HR, 6.6; *p* < 0.001) or after surgery (HR, 13.0; *p* < 0.001). Estimated 3-year recurrence-free survival was 33% for post-operative ctDNA-positive patients and 87% for the postoperative ctDNA-negative patients.	Post-op: 11.9% Post-Treatment: not reported Surveillance: not reported
Tie et al., 2019 [66]	96	III	Safe-SeqS	All patients	Estimated 3-year RFS was 30% when ctDNA was detectable after chemotherapy and 77% when ctDNA was undetectable (HR, 6.8; 95% CI, 11.0–157.0; *p* < 0.001)	Post-op: 21% Post-Treatment: 17% Surveillance: Not tested
Tie et al., 2016 [67]	37	IV (resectable colorectal liver metastases)	Safe-SeqS	70%	ctDNA detectable at a median of 3 months prior to clinical recurrence. Ten of 10 pts (100%) with positive post-treatment (surgery and chemotherapy) ctDNA experienced recurrence vs. 4 of 27 (15%) with negative post-treatment ctDNA (HR, 13.16, *p* < 0.0001)	Post-op: 24.3% Post-Treatment: 27% Surveillance: 32.4%
Khakoo et al., 2020 [68]	47	Locally advanced rectal cancer	ddPCR	91.3%	All 3 patients with detectable ctDNA post-surgery relapsed compared with none of the 20 patients with undetectable ctDNA (*p* = 0.001)	Post-op: 13% Post-Treatment: not reported Surveillance: not reported
Parikh et al., 2019 [69]	72	Stage II–III	Guardant health NGS	41.2%	Patients who were ctDNA-positive after standard therapy completion had a recurrence positive predictive value 93%, negative predictive value 80%, (HR, 11.29; *p* < 0.0001)	Post-op: 19% (surgery arm only) Post-Treatment: 22.2% (chemotherapy arm only) Surveillance: not reported
Overman et al., 2017 [70]	54	IV (resectable liver metastases)	30 kb ctDNA digital sequencing panel (Guardant Health) covering SNVs in 21 genes	Not reported	In 43 patients who underwent successful resection of all visible disease, post-op detection of ctDNA significantly correlated with RFS (HR, 3.1; 95% CI, 1.7–9.1; *p* = 0.002) with 2-year RFS of 0% vs. 47%. ctDNA detected at median of 5.1 months prior to radiographic recurrence.	Post-op: 44% Post-Treatment: Not reported Surveillance: Not reported
Tarazona et al., 2019 [71]	150	Stage II–III	ddPCR	37.2%	Detection of ctDNA after surgery and in serial plasma samples during follow-up were associated with poorer DFS (HR, 17.56; log-rank *p =* 0.0014 and HR, 11.33; log-rank *p =* 0.0001, respectively) In patients treated with adjuvant chemotherapy, presence of ctDNA after therapy was associated with early relapse (HR, 10.02; log-rank *p* < 0.0001)	Post-op: 20.3% Post-Treatment: 28% (patients receiving adjuvant chemotherapy) Surveillance: Not reported

Abbreviations: ctDNA, circulating tumour DNA; CRC, colorectal cancer; HR, hazard ratio; CEA, Carcinoembryonic antigen; ddPCR, droplet digital PCR; CI, confidence interval; DFS, disease-free survival; RFS, recurrence-free survival; NGS, Next-generation sequencing; post-op, post-operative; SNVs, single-nucleotide variant.

**Table 2:** Ongoing ctDNA-based randomised adjuvant trials in non-metastatic colorectal cancer.

Trial Name/Country	Patient Population	Sample Size	ctDNA Assay	Timing of ctDNA Testing	Trial Intervention	Primary Objective
**ctDNA-Guided Strategy Design**						
DYNAMIC (ACTRN-12615000381583) Australia	Stage II colon cancer	450	Safe-SeqS	Week 4 and 7 post-op	**Standard of care:** clinician determined management (surveillance or adjuvant chemotherapy) based on standard clinicopathological features **ctDNA-guided:** ctDNA-positive → adjuvant chemotherapy; ctDNA-negative → surveillance	To demonstrate that an adjuvant therapy strategy based on post-op ctDNA results will reduce the number of patients receiving adjuvant chemotherapy without compromising recurrence-free survival
DYNAMIC-III (ACTRN-12617001566325) Australia/New Zealand	Stage III colon cancer	1000	Safe-SeqS	Week 5–6 post-op	**Standard of care:** clinician determined standard of care adjuvant chemotherapy based on clinical risk **ctDNA-guided:** ctDNA-positive → escalated chemotherapy regimen from pre-planned treatment (increase duration or number of agents); ctDNA-negative → de-escalated chemotherapy regimen from pre-planned treatment (reduction in duration or number of agents)	To evaluate the impact of de-escalation/escalation treatment strategies as informed by post-op ctDNA-informed management Achieve an acceptable rate of de-escalation in the ctDNA-informed negative cohort (phase II) Demonstrate non-inferiority of ctDNA-guided management with respect to recurrence in the de-escalation (ctDNA-informed negative) cohort (phase III) Investigate superiority of a ctDNA-informed management with respect to recurrence in the escalation (ctDNA-informed positive) cohort (Phase III)
DYNAMIC-RECTAL (ACTRN-12617001560381) Australia/New Zealand	Locally advanced rectal cancer	408	Safe-SeqS	Week 4 and 7 post-op	**Standard of care:** clinician determined management (surveillance or adjuvant chemotherapy) based on standard clinicopathological features **ctDNA-guided:** ctDNA-positive → adjuvant chemotherapy; ctDNA-negative and ypN0 → surveillance; ctDNA-negative and ypN+ → surveillance or adjuvant chemotherapy at clinician’s choice	To demonstrate that an adjuvant therapy strategy incorporating ctDNA results in addition to standard pathologic risk assessment will reduce the number of patients receiving adjuvant chemotherapy without compromising recurrence-free survival
TRACC (NCT04050345) [79] United Kingdom	High risk stage II, stage III colorectal cancer	1621	NGS-based 22-gene colorectal panel	<8 weeks post-op, 3 months post-op	**Standard of care:** 6 months of capecitabine or 3 months of CAPOX **ctDNA-guided:** ctDNA-positive → standard adjuvant chemotherapy; ctDNA-negative → de-escalate treatment but re-escalate if ctDNA becomes positive at 3 months	To demonstrate non-inferiority in 3-year DFS between standard of care arm and ctDNA-guided adjuvant chemotherapy arm
MEDOCC-CrEATE (NL6281/NTR6455) [80] Netherlands	Stage II colon cancer	1320	PGDx elio™	4–21 days post-op	**Standard of care:** surveillance **ctDNA-guided:** ctDNA-positive → 6 months of CAPOX; ctDNA-negative → surveillance	To investigate the willingness of patients to receive adjuvant chemotherapy after detection of ctDNA post-surgery
**Marker-by-Treatment Interaction Design**						
NRG GI-005 (COBRA) NCT04068103 [81] United States/Canada	Stage IIA colon cancer	1408	Guardant LUNAR-1™	Post-op	**Standard of care:** Surveillance **ctDNA-guided:** ctDNA-positive → adjuvant FOLFOX/CAPOX; ctDNA-negative → surveillance	To compare the clearance of ctDNA between arms for the baseline ctDNA-positive patient at 6 months (phase II) To compare median RFS between arms for the baseline ctDNA-positive patients at 6 months (phase III)
CIRCULATE AIO-KRK-0217 (NCT04089631) [82] Germany	Stage II colon cancer (MSS tumours)	4812	Not reported	Post-op	ctDNA-positive patients randomised to: **Standard of care:** surveillance **Experimental:** adjuvant chemotherapy (capecitabine or CAPOX)	To compare the disease-free survival in patients who are positive for postoperative ctDNA treated with or without adjuvant chemotherapy
CIRCULATE PRODIGE 70 (NCT04120701) [83] France	Stage II colon cancer	1980	ddPCR (2 methylated markers WIF1 and NPY)	Week 2–8 post-op	198 ctDNA-positive patients randomised to: **Standard of care:** surveillance **Experimental:** adjuvant FOLFOX	To demonstrate a 17.5% gain in 3-year DFS in post-op ctDNA-positive patients treated with adjuvant FOLFOX compared to observation alone
VEGA (UMIN000039205) [84] Japan	High-risk stage II, low-risk stage III colon cancer—ctDNA-negative	1240	Signatera™	1-month post-op	Post-op ctDNA-negative patients randomised to: **Standard of care:** 3 months of CAPOX **Experimental:** Surveillance Patients enroll in ALTAIR study if ctDNA becomes positive at 3 months	To demonstrate the non-inferiority of observation vs. adjuvant CAPOX with absence of ctDNA at 1 month post-surgery
**ctDNA-Enriched 2nd Line Adjuvant Therapy Trial**						
ALTAIR (UMIN000039205) [85] Japan	Stage II/III colorectal cancer or stage IV with resectable metastases	240	Signatera™	1-month post-op and after 3 months of standard adjuvant CAPOX	Patients who are ctDNA-positive after completion of 3 months adjuvant CAPOX are randomised to: **Standard of care:** placebo/surveillance **Experimental:** trifluridine/tipiracil	To demonstrate the superiority of trifluridine/tipiracil over placebo in patients with ctDNA that remains positive after standard adjuvant therapy
ACT-3 (NCT04259944) United States	Stage III colorectal cancer	500	Guardant LUNAR-1™	3–6 weeks post adjuvant chemo	Patients who are ctDNA-positive after completion of 3–6 months of adjuvant FOLFOX/CAPOX are randomised to: **Standard of care:** surveillance **Experimental:** (a) FOLFIRI (MSS/BRAF wild-type) (b) Encorafenib/Binimetinib/Cetuximab (BRAF mutant) (c) Nivolumab (MSI-H)	To demonstrate the superiority of FOLFIRI over surveillance in patients with positive ctDNA after standard adjuvant therapy
**ctDNA-Guided Surveillance Strategy Design**						
IMPROVE-IT2 (NCT04084249) [86] Denmark	High risk stage II, stage III colorectal cancer	254	Droplet digital PCR (colorectal panel)	Every 4 months post-op for 24 months	**Standard of care:** standard Danish follow-up program (CT scans at 12 and 36 months) **ctDNA-guided surveillance:** 3-monthly FDG-PET/CT for patient with positive ctDNA during surveillance	To demonstrate that ctDNA guided post-operative surveillance combining ctDNA and radiological assessments could result in earlier detection of recurrent disease and identify more patients eligible for curative treatment

## References

[1] Bray F., Ferlay J., Soerjomataram I., Siegel R.L., Torre L.A., Jemal A. (2018). Global cancer statistics 2018: GLOBOCAN estimates of incidence and mortality worldwide for 36 cancers in 185 countries. CA Cancer J. Clin..

[2] Douaiher J., Ravipati A., Grams B., Chowdhury S., Alatise O., Are C. (2017). Colorectal cancer-global burden, trends, and geographical variations. J. Surg. Oncol..

[3] Venook A.P., Niedzwiecki N., Lenz H.-J., Innocenti F., Fruth B., Meyerhardt J.A., Schrag D., Greene C., O’Neil B.H., Atkins J.N. (2017). Effect of First-Line Chemotherapy Combined with Cetuximab or Bevacizumab on Overall Survival in Patients with KRAS Wild-Type Advanced or Metastatic Colorectal Cancer: A Randomized Clinical Trial. JAMA.

[4] Cremolini C., Loupakis F., Antoniotti C., Lupi C., Sensi E., Lonardi S., Mezi S., Tomasello G., Ronzoni M., Zaniboni A. (2015). FOLFOXIRI plus bevacizumab versus FOLFIRI plus bevacizumab as first-line treatment of patients with metastatic colorectal cancer: Updated overall survival and molecular subgroup analyses of the open-label, phase 3 TRIBE study. Lancet Oncol..

[5] Heinemann V., von Weikersthal L.F., Decker T., Kiani A., Vehling-Kaiser U., Al-Batran S.-E., Heintges T., Lerchenmüller C., Kahl C., Seipelt G. (2014). FOLFIRI plus cetuximab versus FOLFIRI plus bevacizumab as first-line treatment for patients with metastatic colorectal cancer (FIRE-3): A randomised, open-label, phase 3 trial. Lancet Oncol..

[6] Normanno N., Abate R.E., Lambiase M., Forgione L., Cardone C., Iannaccone A., Sacco A., Rachiglio A., Martinelli E., Rizzi D. (2018). RAS testing of liquid biopsy correlates with the outcome of metastatic colorectal cancer patients treated with first line FOLFIRI plus cetuximab in the CAPRI-GOIM trial. Ann. Oncol..

[7] Vidal J., Muinelo L., Dalmases A., Jones F., Edelstein D., Iglesias M., Orrillo M., Abalo A., Rodríguez C., Brozos E. (2017). Plasma ctDNA RAS mutation analysis for the diagnosis and treatment monitoring of metastatic colorectal cancer patients. Ann. Oncol..

[8] Grasselli J., Elez E., Caratù G., Matito J., Santos C., Macarulla T., Vidal J., Garcia M., Viéitez J., Paéz D. (2017). Concordance of blood- and tumor-based detection of RAS mutations to guide anti-EGFR therapy in metastatic colorectal cancer. Ann. Oncol..

[9] Sargent D., Sobrero A., Grothey A., O’Connell M.J., Buyse M., Andre T., Zheng Y., Green E., Labianca R., O’Callaghan C. (2009). Evidence for Cure by Adjuvant Therapy in Colon Cancer: Observations Based on Individual Patient Data From 20,898 Patients on 18 Randomized Trials. J. Clin. Oncol..

[10] Shah M.A., Renfro L.A., Allegra C.J., André T., de Gramont A., Schmoll H.-J., Haller D.G., Alberts S.R., Yothers G., Sargent D.J. (2016). Impact of Patient Factors on Recurrence Risk and Time Dependency of Oxaliplatin Benefit in Patients with Colon Cancer: Analysis from Modern-Era Adjuvant Studies in the Adjuvant Colon Cancer End Points (ACCENT) Database. J. Clin. Oncol..

[11] Labianca R., Nordlinger B., Beretta G.D., Mosconi S., Mandalà M., Cervantes A., Arnold D. (2013). Early colon cancer: ESMO Clinical Practice Guidelines for diagnosis, treatment, and follow-up. Ann. Oncol..

[12] Argilés G., Tabernero J., Labianca R., Hochhauser D., Salazar R., Iveson T., Laurent-Puig P., Quirke P., Yoshino T., Taieb J. (2020). Localised colon cancer: ESMO Clinical Practice Guidelines for diagnosis, treatment, and follow-up. Ann. Oncol..

[13] Costas-Chavarri A., Temin S., Shah M.A. (2019). Treatment of Patients with Early-Stage Colorectal Cancer: ASCO Resource-Stratified Guideline Summary. J. Oncol. Pr..

[14] O’Connor E.S., Greenblatt D.Y., LoConte N.K., Gangnon R.E., Liou J.-I., Heise C.P., Smith M.A. (2011). Adjuvant Chemotherapy for Stage II Colon Cancer with Poor Prognostic Features. J. Clin. Oncol..

[15] Figueredo A., Charette M.L., Maroun J., Brouwers M.C., Zuraw L. (2004). Adjuvant Therapy for Stage II Colon Cancer: A Systematic Review from the Cancer Care Ontario Program in Evidence-Based Care’s Gastrointestinal Cancer Disease Site Group. J. Clin. Oncol..

[16] André T., Boni C., Navarro M., Tabernero J., Hickish T., Topham C., Bonetti A., Clingan P., Bridgewater J., Rivera F. (2009). Improved Overall Survival with Oxaliplatin, Fluorouracil, and Leucovorin As Adjuvant Treatment in Stage II or III Colon Cancer in the MOSAIC Trial. J. Clin. Oncol..

[17] André T., de Gramont A.A., Vernerey D., Chibaudel B.B., Bonnetain F., Tijeras-Raballand A.A., Scriva A.A., Hickish T.T., Tabernero J., van Laethem J.L. (2015). Adjuvant Fluorouracil, Leucovorin, and Oxaliplatin in Stage II to III Colon Cancer: Updated 10-Year Survival and Outcomes According to BRAF Mutation and Mismatch Repair Status of the MOSAIC Study. J. Clin. Oncol..

[18] Schmoll H.-J., Tabernero J., Maroun J., de Braud F., Price T., Van Cutsem E., Hill M., Hoersch S., Rittweger K., Haller D.G. (2015). Capecitabine Plus Oxaliplatin Compared with Fluorouracil/Folinic Acid as Adjuvant Therapy for Stage III Colon Cancer: Final Results of the NO16968 Randomized Controlled Phase III Trial. J. Clin. Oncol..

[19] Kidwell K.M., Yothers G., Ganz P.A., Land S.R., Ko C.Y., Cecchini R.S., Kopec J.A., Wolmark N. (2012). Long-term neurotoxicity effects of oxaliplatin added to fluorouracil and leucovorin as adjuvant therapy for colon cancer: Results from National Surgical Adjuvant Breast and Bowel Project trials C-07 and LTS-01. Cancer.

[20] Mols F., Beijers T., Lemmens V., Hurk C.J.V.D., Vreugdenhil G., van de Poll-Franse L.V. (2013). Chemotherapy-Induced Neuropathy and Its Association with Quality of Life Among 2- to 11-Year Colorectal Cancer Survivors: Results from the Population-Based PROFILES Registry. J. Clin. Oncol..

[21] Pachman D.R., Qin R., Seisler D.K., Smith E.M., Beutler A.S., Ta L.E., Lafky J.M., Wagner-Johnston N.D., Ruddy K.J., Dakhil S.R. (2015). Clinical Course of Oxaliplatin-Induced Neuropathy: Results from the Randomized Phase III Trial N08CB (Alliance). J. Clin. Oncol..

[22] Grothey A., Sobrero A., Shields A.F., Yoshino T., Paul J., Taieb J., Souglakos J., Shi Q., Kerr R., Labianca R. (2018). Duration of Adjuvant Chemotherapy for Stage III Colon Cancer. N. Engl. J. Med..

[23] Sobrero A.F., Andre T., Meyerhardt J.A., Grothey A., Iveson T., Yoshino T., Sougklakos I., Meyers J.P., Labianca R., Saunders M.P. (2020). Overall survival (OS) and long-term disease-free survival (DFS) of three versus six months of adjuvant (adj) oxaliplatin and fluoropyrimidine-based therapy for patients (pts) with stage III colon cancer (CC): Results from the IDEA (International Duration Evaluation of Adj chemotherapy) collaboration. J. Clin. Oncol..

[24] Saltz L., Niedzwiecki D., Hollis D., Goldberg R.M., Hantel A., Thomas J.P., Fields A.L., Mayer R.J. (2007). Irinotecan Fluorouracil Plus Leucovorin Is Not Superior to Fluorouracil Plus Leucovorin Alone as Adjuvant Treatment for Stage III Colon Cancer: Results of CALGB 89803. J. Clin. Oncol..

[25] van Cutsem E., Labianca R., Bodoky G., Barone C., Aranda E., Nordlinger B., Topham C., Tabernero J., André T., Sobrero A.F. (2009). Randomized Phase III Trial Comparing Biweekly Infusional Fluorouracil/Leucovorin Alone or With Irinotecan in the Adjuvant Treatment of Stage III Colon Cancer: PETACC-3. J. Clin. Oncol..

[26] Ychou M., Raoul J.-L., Douillard J.-Y., Gourgou-Bourgade S., Bugat R., Mineur L., Viret F., Becouarn Y., Bouché O., Gamelin E. (2009). A phase III randomised trial of LV5FU2 + irinotecan versus LV5FU2 alone in adjuvant high-risk colon cancer (FNCLCC Accord02/FFCD9802). Ann. Oncol..

[27] Sargent D.J., Alberts S.R., Nair S., Mahoney M.R., Mooney M., Thibodeau S.N., Smyrk T.C., Sinicrope F.A., Chan E., Gill S. (2012). Effect of Oxaliplatin, Fluorouracil, and Leucovorin With or Without Cetuximab on Survival Among Patients with Resected Stage III Colon Cancer. JAMA.

[28] Taieb J., Balogoun R., le Malicot K., Tabernero J., Mini E., Folprecht G., van Laethem J.-L., Emile J.-F., Mulot C., Fratté S. (2017). Adjuvant FOLFOX +/− cetuximab in fullRAS andBRAF wildtype stage III colon cancer patients. Ann. Oncol..

[29] de Gramont A., van Cutsem E., Schmoll H.-J., Tabernero J., Clarke S., Moore M.J., Cunningham D., Cartwright T.H., Hecht J.R., Rivera F. (2012). Bevacizumab plus oxaliplatin-based chemotherapy as adjuvant treatment for colon cancer (AVANT): A phase 3 randomised controlled trial. Lancet Oncol..

[30] Kerr R., Love S., Segelov E., Johnstone E., Falcon B., Hewett P., Weaver A., Church D., Scudder C., Pearson S. (2016). Adjuvant capecitabine plus bevacizumab versus capecitabine alone in patients with colorectal cancer (QUASAR 2): An open label, randomised phase 3 trial. Lancet Oncol..

[31] Jahr S., Hentze H., Englisch S., Hardt D., Fackelmayer F.O., Hesch R.D., Knippers R. (2001). DNA fragments in the blood plasma of cancer patients: Quantitations and evidence for their origin from apoptotic and necrotic cells. Cancer Res..

[32] Volik S., Alcaide M., Morin R.D., Collins C.C. (2016). Cell-free DNA (cfDNA): Clinical Significance and Utility in Cancer Shaped by Emerging Technologies. Mol. Cancer Res..

[33] Mandel P., Metais P. (1948). Nuclear Acids in Human Blood Plasma. C. R. Seances Soc. Biol. Fil..

[34] Vasioukhin V., Anker P., Maurice P., Lyautey J., Lederrey C., Stroun M. (1994). Point mutations of the N-ras gene in the blood plasma DNA of patients with myelodysplastic syndrome or acute myelogenous leukaemia. Br. J. Haematol..

[35] Bettegowda C., Sausen M., Leary R.J., Kinde I., Wang Y., Agrawal N., Bartlett B.R., Wang H., Luber B., Alani R.M. (2014). Detection of Circulating Tumor DNA in Early- and Late-Stage Human Malignancies. Sci. Transl. Med..

[36] Bachet J., Bouché O., Taïeb J., Dubreuil O., Garcia M., Meurisse A., Normand C., Gornet J., Artru P., Louafi S. (2018). RAS mutation analysis in circulating tumor DNA from patients with metastatic colorectal cancer: The AGEO RASANC prospective multicenter study. Ann. Oncol..

[37] Diehl F., Schmidt K., Choti M.A., E Romans K., Goodman S.N., Li M., Thornton K., Agrawal N., Sokoll L.J., Szabo S.A. (2008). Circulating mutant DNA to assess tumor dynamics. Nat. Med..

[38] Osumi H., Shinozaki E., Yamaguchi K., Zembutsu H. (2019). Early change in circulating tumor DNA as a potential predictor of response to chemotherapy in patients with metastatic colorectal cancer. Sci. Rep..

[39] Tie J., Kinde I., Wang Y., Wong H.L., Roebert J., Christie M., Tacey M., Wong R., Singh M., Karapetis C.S. (2015). Circulating tumor DNA as an early marker of therapeutic response in patients with metastatic colorectal cancer. Ann. Oncol..

[40] Buccisano F., Maurillo L., del Principe M.I., del Poeta G., Sconocchia G., Lo-Coco F., Arcese W., Amadori S., Venditti A. (2012). Prognostic and therapeutic implications of minimal residual disease detection in acute myeloid leukemia. Blood.

[41] Borowitz M.J., Devidas M., Hunger S.P., Bowman W.P., Carroll A.J., Carroll W.L., Linda S., Martin P.L., Pullen D.J., Viswanatha D. (2008). Clinical significance of minimal residual disease in childhood acute lymphoblastic leukemia and its relationship to other prognostic factors: A Children’s Oncology Group study. Blood.

[42] Brüggemann M., Raff T., Flohr T., Gökbuget N., Nakao M., Droese J., Lüschen S., Pott C., Ritgen M., Scheuring U. (2006). Clinical significance of minimal residual disease quantification in adult patients with standard-risk acute lymphoblastic leukemia. Blood.

[43] Landgren O., Devlin S., Boulad M., Mailankody S. (2016). Role of MRD status in relation to clinical outcomes in newly diagnosed multiple myeloma patients: A meta-analysis. Bone Marrow Transplant..

[44] Landgren O., Lu S.X., Hultcrantz M. (2018). MRD Testing in Multiple Myeloma: The Main Future Driver for Modern Tailored Treatment. Semin. Hematol..

[45] Kwok M., Rawstron A.C., Varghese A., Evans P.A.S., O’Connor S.J.M., Doughty C., Newton D.J., Moreton P., Hillmen P. (2016). Minimal residual disease is an independent predictor for 10-year survival in CLL. Blood.

[46] Hoelzer D., Bassan R., Dombret H., Fielding A., Ribera J.M., Buske C. (2016). Acute lymphoblastic leukaemia in adult patients: ESMO Clinical Practice Guidelines for diagnosis, treatment and follow-up. Ann. Oncol..

[47] Gorgannezhad L., Umer M., Islam N., Nguyen N.-T., Shiddiky M.J. (2018). Circulating tumor DNA and liquid biopsy: Opportunities, challenges, and recent advances in detection technologies. Lab Chip.

[48] Wan J.C.M., Massie C., Garcia-Corbacho J., Mouliere F., Brenton J.D., Caldas C., Pacey S., Baird C.C.S.P.R., Rosenfeld N. (2017). Liquid biopsies come of age: Towards implementation of circulating tumour DNA. Nat. Rev. Cancer.

[49] Merker J.D., Oxnard G.R., Compton C., Diehn M., Hurley P., Lazar A.J., Lindeman N., Lockwood C.M., Rai A.J., Schilsky R.L. (2018). Circulating Tumor DNA Analysis in Patients with Cancer: American Society of Clinical Oncology and College of American Pathologists Joint Review. Arch. Pathol. Lab. Med..

[50] Perakis S., Auer M., Belic J., Heitzer E. (2017). Advances in Circulating Tumor DNA Analysis. Advances in Clinical Chemistry.

[51] Dasari A., Morris V.K., Allegra C.J., Atreya C., Benson A.B., Boland P., Chung K., Copur M.S., Corcoran R.B., Deming D.A. (2020). ctDNA applications and integration in colorectal cancer: An NCI Colon and Rectal–Anal Task Forces whitepaper. Nat. Rev. Clin. Oncol..

[52] Kinde I., Wu J., Papadopoulos N., Kinzler K.W., Vogelstein B. (2011). Detection, and quantification of rare mutations with massively parallel sequencing. Proc. Natil. Acad. Sci. USA.

[53] Newman A.M., Lovejoy A.F., Klass D.M., Kurtz D.M., Chabon J.J., Scherer F., Stehr H., Liu C.L., Bratman S.V., Say C. (2016). Integrated digital error suppression for improved detection of circulating tumor DNA. Nat. Biotechnol..

[54] Forshew T., Murtaza M., Parkinson C., Gale D., Tsui D.W.Y., Kaper F., Dawson S.-J., Piskorz A.M., Jimenez-Linan M., Bentley D. (2012). Noninvasive Identification and Monitoring of Cancer Mutations by Targeted Deep Sequencing of Plasma DNA. Sci. Transl. Med..

[55] McDonald B.R., Contente-Cuomo T., Sammut S.-J., Odenheimer-Bergman A., Ernst B., Perdigones N., Chin S.-F., Farooq M., Mejia R., Cronin P.A. (2019). Personalized circulating tumor DNA analysis to detect residual disease after neoadjuvant therapy in breast cancer. Sci. Transl. Med..

[56] Reinert T., Henriksen T.V., Christensen E., Sharma S., Salari R., Sethi H., Knudsen M., Nordentoft I., Wu H.-T., Tin A.S. (2019). Analysis of Plasma Cell-Free DNA by Ultradeep Sequencing in Patients with Stages I to III Colorectal Cancer. JAMA Oncol..

[57] Abbosh C., Frankell A., Garnett A., Harrison T., Weichert M., Licon A., Veeriah S., Daber B., Moreau M., Chesh A. (2020). Abstract CT023: Phylogenetic tracking and minimal residual disease detection using ctDNA in early-stage NSCLC: A lung TRACERx study. Proceedings of the Tumor Biology.

[58] Heider K., Gale D., Ruiz-Valdepenas A., Marsico G., Sharma G., Perry M., Osborne R., Howarth K., Lazarus T., Rundell V. (2020). Abstract 735: Sensitive detection of ctDNA in early-stage non-small cell lung cancer patients with a personalized sequencing assay. Proceedings of the Clinical Trials.

[59] Postel M., Roosen A., Laurent-Puig P., Taly V., Wang-Renault S.-F. (2018). Droplet-based digital PCR and next generation sequencing for monitoring circulating tumor DNA: A cancer diagnostic perspective. Expert Rev. Mol. Diagn..

[60] Hindson B.J., Ness K.D., Masquelier D.A., Belgrader P., Heredia N.J., Makarewicz A.J., Bright I.J., Lucero M.Y., Hiddessen A.L., Legler T.C. (2011). High-Throughput Droplet Digital PCR System for Absolute Quantitation of DNA Copy Number. Anal. Chem..

[61] Commissioner of the U.S. Food and Drug Administration. https://www.fda.gov/home.

[62] Tie J., Wang Y., Tomasetti C., Li L., Springer S., Kinde I., Silliman N., Tacey M., Wong H.-L., Christie M. (2016). Circulating tumor DNA analysis detects minimal residual disease and predicts recurrence in patients with stage II colon cancer. Sci. Transl. Med..

[63] Schøler L.V., Reinert T., Ørntoft M.-B.W., Kassentoft C.G., Árnadóttir S.S., Vang S., Nordentoft I., Knudsen M., Lamy P., Andreasen D. (2017). Clinical Implications of Monitoring Circulating Tumor DNA in Patients with Colorectal Cancer. Clin. Cancer Res..

[64] Taieb J., Taly V., Vernerey D., Bourreau C., Bennouna J., Faroux R., Desrame J., Bouche O., Borg C., Egreteau J. (2019). Analysis of circulating tumour DNA (ctDNA) from patients enrolled in the IDEA-FRANCE phase III trial: Prognostic and predictive value for adjuvant treatment duration. Ann. Oncol..

[65] Tie J., Cohen J.D., Wang Y., Li L., Christie M., Simons K., Elsaleh H., Kosmider S., Wong R., Yip D. (2019). Serial circulating tumour DNA analysis during multimodality treatment of locally advanced rectal cancer: A prospective biomarker study. Gut.

[66] Tie J., Cohen J.D., Wang Y., Christie M., Simons K., Lee M., Wong R., Kosmider S., Ananda S., McKendrick J. (2019). Circulating Tumor DNA Analyses as Markers of Recurrence Risk and Benefit of Adjuvant Therapy for Stage III Colon Cancer. JAMA Oncol..

[67] Tie J., Wang Y., Springer S., Kinde I., Wong H.-L., Kosmider S., Tran B., Christie M., Thomson B.N., Wong R. (2016). Serial circulating tumor DNA (ctDNA) and recurrence risk in patients (pts) with resectable colorectal liver metastasis (CLM). J. Clin. Oncol..

[68] Khakoo S., Carter P.D., Brown G., Valeri N., Picchia S., Bali M.A., Shaikh R., Jones T., Begum R., Rana I. (2020). MRI Tumor Regression Grade and Circulating Tumor DNA as Complementary Tools to Assess Response and Guide Therapy Adaptation in Rectal Cancer. Clin. Cancer Res..

[69] Parikh A.R., van Seventer E.E., Boland G.M., Hartwig A., Jaimovich A., Raymond V.M., Talasaz A., Corcoran R.B. (2019). A plasma-only integrated genomic and epigenomic circulating tumor DNA (ctDNA) assay to inform recurrence risk in colorectal cancer (CRC). J. Clin. Oncol..

[70] Overman M.J., Vauthey J.-N., Aloia T.A., Conrad C., Chun Y.S., Pereira A., Jiang Z., Crosby S., Wei S., Raghav K.P.S. (2017). Circulating tumor DNA (ctDNA) utilizing a high-sensitivity panel to detect minimal residual disease post liver hepatectomy and predict disease recurrence. J. Clin. Oncol..

[71] Cervantes A., Gimeno-Valiente F., Gambardella V., Zuñiga S., Rentero-Garrido P., Huerta M., Roselló S., Martinez-Ciarpaglini C., Carbonell-Asins J., Carrasco F. (2019). Targeted next-generation sequencing of circulating-tumor DNA for tracking minimal residual disease in localized colon cancer. Ann. Oncol..

[72] Meyerhardt J.A., Mangu P.B., Flynn P.J., Korde L., Loprinzi C.L., Minsky B.D., Petrelli N.J., Ryan K., Schrag D.H., Wong S.L. (2013). Follow-Up Care, Surveillance Protocol, and Secondary Prevention Measures for Survivors of Colorectal Cancer: American Society of Clinical Oncology Clinical Practice Guideline Endorsement. J. Clin. Oncol..

[73] Primrose J.N., Perera R., Gray A., Rose P., Fuller A., Corkhill A., George S., Mant D. (2014). Effect of 3 to 5 Years of Scheduled CEA and CT Follow-up to Detect Recurrence of Colorectal Cancer. JAMA.

[74] Rosati G., Ambrosini G., Barni S., Andreoni B., Corradini G., Luchena G., Daniele B., Gaion F., Oliverio G., Duro M. (2016). A randomized trial of intensive versus minimal surveillance of patients with resected Dukes B2-C colorectal carcinoma. Ann. Oncol..

[75] Lepage C., Phelip J., Cany L., Barbier E., Manfredi S., Deguiral P., Faroux R., Baconnier M., Pezet D., Duchmann J. (2020). Effect of 5 years of imaging and CEA follow-up to detect recurrence of colorectal cancer (CRC)—PRODIGE 13 a FFCD phase III trial. Ann. Oncol..

[76] Litvak A., Cercek A., Segal N., Reidy-Lagunes D., Stadler Z.K., Yaeger R.D., Kemeny N.E., Weiser M.R., Pessin M.S., Saltz L. (2014). False-Positive Elevations of Carcinoembryonic Antigen in Patients with a History of Resected Colorectal Cancer. J. Natl. Compr. Cancer Netw..

[77] Newton K.F., Newman W., Hill J. (2011). Review of biomarkers in colorectal cancer. Color. Dis..

[78] Goldstein M.J., Mitchell E.P. (2005). Carcinoembryonic Antigen in the Staging and Follow-up of Patients with Colorectal Cancer. Cancer Investig..

[79] Anandappa G., Starling N., Peckitt C., Bryant A., Begum R., Carter P., Hatt S., Khakoo S.S., Turner A., Kidd S. (2020). TRACC: Tracking mutations in cell-free DNA to predict relapse in early colorectal cancer—A randomized study of circulating tumour DNA (ctDNA) guided adjuvant chemotherapy versus standard of care chemotherapy after curative surgery in patients with high-risk stage II or stage III colorectal cancer (CRC). J. Clin. Oncol..

[80] Schraa S.J., Van Rooijen K.L., Van Der Kruijssen D.E.W., Alarcón C.R., Phallen J., Sausen M., Simmons J., Coupé V.M.H., Van Grevenstein W.M.U., on behalf of the PLCRC-MEDOCC group (2020). Circulating tumor DNA guided adjuvant chemotherapy in stage II colon cancer (MEDOCC-CrEATE): Study protocol for a trial within a cohort study. BMC Cancer.

[81] Morris V.K., Yothers G., Kopetz S., Jacobs S.A., Lucas P.C., Iqbal A., Boland P.M., Deming D.A., Scott A.J., Lim H.J. (2020). Phase II/III study of circulating tumor DNA as a predictive biomarker in adjuvant chemotherapy in patients with stage II colon cancer: NRG-GI005 (COBRA). J. Clin. Oncol..

[82] Folprecht G., Reinacher-Schick A., Tannapfel A., Weitz J., Kossler T., Weiss L., Aust D.E., Von Bubnoff N., Kramer M., Thiede C. (2020). Circulating tumor DNA-based decision for adjuvant treatment in colon cancer stage II evaluation: (CIRCULATE-trial) AIO-KRK-0217. J. Clin. Oncol..

[83] Taieb J., Benhaim L., Puig P.L., le Malicot K., Emile J.F., Geillon F., Tougeron D., Manfredi S., Chauvenet M., Taly V. (2020). Decision for adjuvant treatment in stage II colon cancer based on circulating tumor DNA:The CIRCULATE-PRODIGE 70 trial. Dig. Liver Dis..

[84] Yukami H., Mishima S., Kotani D., Oki E., Taniguchi H., Nakamura Y., Kato T., Takemasa I., Yamanaka T., Shirasu H. (2020). P-120 Prospective observational study monitoring circulating tumor DNA in resectable colorectal cancer patients undergoing radical surgery: GALAXY study in CIRCULATE-Japan (trial in progress). Ann. Oncol..

[85] Yukami H., Saori M., Kotani D., Oki E., Taniguchi H., Nakamura Y., Kato T., Takemasa I., Yamanaka T., Shirasu H. (2020). 113TiP Prospective observational study monitoring circulating tumour DNA in resectable colorectal cancer patients undergoing radical surgery: GALAXY study in CIRCULATE-Japan. Ann. Oncol..

[86] Nors J., Henriksen T.V., Gotschalck K.A., Juul T., Søgaard J., Iversen L.H., Andersen C.L. (2020). IMPROVE-IT2: Implementing noninvasive circulating tumor DNA analysis to optimize the operative and postoperative treatment for patients with colorectal cancer—Intervention trial 2. Study protocol. Acta Oncol..

[87] Sargent D.J., Conley B.A., Allegra C., Collette L. (2005). Clinical Trial Designs for Predictive Marker Validation in Cancer Treatment Trials. J. Clin. Oncol..

[88] Lonardi S., Montagut C., Pietrantonio F., Elez E., Sartore-Bianchi A., Tarazona N., Sciallero S., Zampino M.G., Mosconi S., Muñoz S. (2020). The PEGASUS trial: Post-surgical liquid biopsy-guided treatment of stage III and high-risk stage II colon cancer patients. J. Clin. Oncol..

[89] Tie J., Cohen J.D., Lo S.N., Wang Y., Li L., Christie M., Lee M., Wong R., Kosmider S., Skinner I. (2021). Prognostic significance of postsurgery circulating tumor DNA in nonmetastatic colorectal cancer: Individual patient pooled analysis of three cohort studies. Int. J. Cancer.

[90] Gai W., Sun K. (2019). Epigenetic Biomarkers in Cell-Free DNA and Applications in Liquid Biopsy. Genes.

[91] Schwarzenbach H., Nishida N., Calin G.A., Pantel K. (2014). Clinical relevance of circulating cell-free microRNAs in cancer. Nat. Rev. Clin. Oncol..

[92] Henriksen T.V., Reinert T., Christensen E., Sethi H., Birkenkamp-Demtröder K., Gögenur M., Gögenur I., Zimmermann B.G., Dyrskjøt L., Andersen C.L. (2020). The effect of surgical trauma on circulating free DNA levels in cancer patients—Implications for studies of circulating tumor DNA. Mol. Oncol..

[93] Kasi P.M., Dayyani F., Morris V.K., Kopetz S., Parikh A.R., Starr J.S., Cohen S., Grothey A., Lieu C.H., O’Hara M.H. (2020). Tumor-informed assessment of molecular residual disease and its incorporation into practice for patients with early and advanced-stage colorectal cancer (CRC-MRD Consortia). J. Clin. Oncol..

[94] Garcia-Murillas I., Chopra N., Comino-Méndez I., Beaney M., Tovey H., Cutts R.J., Swift C., Kriplani D., Afentakis M., Hrebien S. (2019). Assessment of Molecular Relapse Detection in Early-Stage Breast Cancer. JAMA Oncol..

[95] Gray R., Quirke P., Handley K., Lopatin M., Magill L., Baehner F.L., Beaumont C., Clark-Langone K.M., Yoshizawa C.N., Lee M. (2011). Validation Study of a Quantitative Multigene Reverse Transcriptase–Polymerase Chain Reaction Assay for Assessment of Recurrence Risk in Patients with Stage II Colon Cancer. J. Clin. Oncol..

[96] Niedzwiecki D., Frankel W.L., Venook A.P., Ye X., Friedman P.N., Goldberg R.M., Mayer R.J., Colacchio T.A., Mulligan J.M., Davison T.S. (2016). Association Between Results of a Gene Expression Signature Assay and Recurrence-Free Interval in Patients with Stage II Colon Cancer in Cancer and Leukemia Group B 9581 (Alliance). J. Clin. Oncol..

[97] Pagès F., Mlecnik B., Marliot F., Bindea G., Ou F.-S., Bifulco C., Lugli A., Zlobec I., Rau T.T., Berger M.D. (2018). International validation of the consensus Immunoscore for the classification of colon cancer: A prognostic and accuracy study. Lancet.

[98] Mlecnik B., Bifulco C., Bindea G., Marliot F., Lugli A., Lee J.J., Zlobec I., Rau T.T., Berger M.D., Nagtegaal I.D. (2020). Multicenter International Society for Immunotherapy of Cancer Study of the Consensus Immunoscore for the Prediction of Survival and Response to Chemotherapy in Stage III Colon Cancer. J. Clin. Oncol..

[99] Galon J., Hermitte F., Mlecnik B., Lugli A., Bifulco C.B., Nagtegaal I.D., Hartmann A., Marliot F., Eynde M.V.D., Roehrl M.H.A. (2020). Immunoscore as a parameter predicting time to recurrence and disease-free survival in T4N0 stage II colon cancer patients. J. Clin. Oncol..

